# Practical Notes and Formulæ

**Published:** 1886-04-20

**Authors:** 


### PRACTICAL NOTES AND FORMULÆ.

**The Treatment of Cholera Infantum.**—(Medical and Sur. Reporter) Dr. J. H. Cartser gives us the following formulæ in the Archieves of Pedriatics: For the vomiting:

℞. Tinct. iodi................................gtt. x,
Aquæ................................℥ ij.

M. Teaspoonful every half hour.

In some cases he uses a powder composed of

℞. Subnitrate of bismuth ........................ʒ i,

Dover's powder..............................grs. iij.

To be divided into twelve powders, one every two hours.

This often does in mild cases. Sometimes, however, if there seems to be a malarial complication he gives quinine, for instance something like this:

℞. Sulphate of quinine........................grs. x,
Tincture of opium..........................gtts. xx,
Syrup yerba santa..........................℥ ij.

M. S. Teaspoonful every two hours.

**Menthol Disks for Sick Headache.**—At a recent meeting of the Societe de Therapeutique, M. Mayet, stated that he had had disks made after the following formula:

℞. Menthol................................grs. vijss,
Chloral hyd..............................grs. vijss,
Spermaceti..............................grs. xxx,
Cocoa-butter............................grs. xv.

A disk is to be bound on over the seat of the pain. Mayet said that he had never observed that these disks had an irritating effect.

**Bichloride of Mercury in Phthisis.**—(Medical News, Phil.) Dr. Kolloch reports that the use of the following prescription in thirty-three cases of pulmonary phthisis invariably, in any stage, produced marked improvement:

℞. Hydrarg bichloride..........................gr. j,
Ammonium chloride..........................ʒ ijss,
Alcohol....................................℥ ij,
Glycerin and water, ad.....................℥ iv.

**Typhoid Fever.**—(Indiana Medical Journal) Dr. John C. Pearson (Lancet) is convinced that many cases of typhoid fever can be lessened in severity and duration by the early and persistent administration of the solution of chlorinated soda. He generally orders:

℞. Sodæ chlorate.............................ʒ iss,

Aquæ......................................℥ vi.

M. Sig. A tablespoonful every three hours.

**Irritability of the Bladder.**—Dr. A. F. Erich, in Phil. Med. Times, says that irritability of the bladder, accompanied by alkaline urine, can be immediately relieved by—

℞. Acidi benzoici.................................ʒ j,

Sodii biboratis.................................ʒ iss,

Aquæ.................................℥ xi.

M. Sig. A teaspoonful every three hours.

**Croup.**—(Indiana Medical Journal.)

℞. Tinct. veratri viridi.................................*m* x,

Syrupi ipecac.................................ʒ j,

Spts. ætheris nitrosi.................................ʒ iij,

Syr. limonis.................................ʒ vi.

M. Sig. One teaspoonful every half hour.

**Ague.**—(Medical World.)

℞. Ammonii iodi.................................ʒ j,

Liq. arsenicalis.................................ʒ ss,

Tinct. calumbæ.................................℥ ss,

Aq. dest.................................℥ iss.

Mix. One teaspoonful three times a day, when the spleen is enlarged.

℞. Quiniæ sulphat.................................ʒ j,

Potass. bromidi.................................ʒ iss,

Acid. suph. dil.................................ʒ iss,

Aq. dest. et syr. simpl. ad.................................℥ iv.

Mix. One tablespoonful three times a day.

℞. Quiniæ sulphat.................................ʒ ss,

Potass. iodidi.................................ʒ ss,

Sodæ salicylatis.................................ʒ j,

Pulv. tragacanth. co.................................ʒ iss,

Aq. chloroformi et aquæ dest. ad.................................℥ viij.

Mix. Sig. Shake the bottle. Two tablespoonfuls three times a day.

**Amenorrhœa.**—(Medical World.)

℞. Potassæ permanangatis, gr. i, or gr. ii, three times a day in pill.

℞. Tint. acteæ racemosæ.................................ʒ j,

Tinct. aconiti.................................*m* xvi,

Aq. dest., ad.................................℥ ij.

Mix. One teaspoonful every hour, with hot mustard pediluvia at bedtime.

**Brown's Iron Bitters.**—(Medical World.) Contains in each fluidrachm:

℞. Iron.......................................gr. i,
Calisaya bark...... ...........................grs. ij,
Phosphorus....... ...........................gr. 1-200,
Coca.......................................gr. i,
Viburnum prunifol..............................gr. i.

**Thompson's Eye Water.**—(Medical World.)

℞. Sulphate of copper..........................grs. x,
Sulphate of zinc.. ...........................grs. xl,
Tincture of saffron...........................ʒ iv,
Tincture of camphor...........................ʒ iv,
Rose water..................................O. ii.

Mix and filter.

**Resorcin in the Treatment of Gonorrhœa.**—Dellerbaugh (Revue de Therapeutique) recommends the use of resorcin combined as follows, in injections, for the cure of gonorrhœa:

℞. Zinc.a cetatis.......... ...............gr. $\frac{1}{4}$ to gr. $\frac{1}{2}$,

Acidi boracici..........................gr. xx,

Resorcin................................ʒ j,

Aquæ dest...............................f ℥ iv.

M. Sig. Inject two drams three times a day.

**Catarrh.**—Dr. De Lamater (in New England Medical Monthly) says: The following has proven so effectual in my practice for catarrh and acute affections of the throat and nasal passages, I wish to give it to the readers of your valuable journal:

℞. Chlorate potash..............................℥ v,

Nitrate potash................................℥ iii,

Carbolic acid (crystal)........................ʒ ii,

Pulv. sach. alb................................℥ ii.

M. Sig. One teaspoonful in a glass of water. Use as gargle or spray in throat and nostrils every three or four hours.

**To Prevent Pitting in Small-Pox.**—

℞. White lead, linseed oil, each in quantity sufficient to make when rubbed together a cream-like paste, to which add 5 per cent. of carbolic acid. Keep the parts freely anointed.

**For Removing Warts.**—Dr. D. C. Platt (in New England Medical Monthly) says: The following has served a good purpose for removing warts. I have found that sometimes it is extremely difficult to get rid of them, but with this wash I am confident of success:

℞. Cryst. argenti nitras.........................℥ j,

Acid nitro mur................................℥ j.

M. Paint with a camel's hair brush once a day. It will usually cure in four days.

